# Serum Urokinase-Type Plasminogen Activator Receptor Does Not Outperform C-Reactive Protein and Procalcitonin as an Early Marker of Severity of Acute Pancreatitis

**DOI:** 10.3390/jcm7100305

**Published:** 2018-09-27

**Authors:** Witold Kolber, Beata Kuśnierz-Cabala, Paulina Dumnicka, Małgorzata Maraj, Małgorzata Mazur-Laskowska, Michał Pędziwiatr, Piotr Ceranowicz

**Affiliations:** 1 Department of Surgery, Complex of Health Care Centers in Wadowice, Karmelicka 5 St., 34-100 Wadowice, Poland; wkolber@wp.pl; 2 Department of Diagnostics, Chair of Clinical Biochemistry, Faculty of Medicine, Jagiellonian University Medical College, Kopernika 15A St., 31-501 Krakow, Poland; 3 Department of Medical Diagnostics, Faculty of Pharmacy, Jagiellonian University Medical College, Medyczna 9 St., 30-688 Krakow, Poland; paulina.dumnicka@uj.edu.pl; 4 Department of Physiology, Faculty of Medicine, Jagiellonian University Medical College, Grzegorzecka 16 St., 31-531 Krakow, Poland; malgorzata.maraj@gmail.com (M.M.); mpcerano@cyf-kr.edu.pl (P.C.); 5 Diagnostics Department of University Hospital in Krakow, Kopernika 15B St., 31-501 Krakow, Poland; mbmazur@cyf-kr.edu.pl; 6 2nd Department of Surgery, Faculty of Medicine, Jagiellonian University Medical College, Kopernika 21 St., 31-501 Krakow, Poland; mpedziwiatr@gmail.com

**Keywords:** urokinase-type plasminogen activator receptor, interleukin 6, early prediction of acute pancreatitis severity, acute kidney injury, liver failure

## Abstract

Severe acute pancreatitis (SAP) concerns 10–20% of acute pancreatitis (AP) patients and is associated with a poor prognosis and high mortality. An early prognosis of the unfavorable outcome, transfer to an intensive care unit (ICU) and the introduction of an adequate treatment are crucial for patients’ survival. Recently, the elevated circulating urokinase-type plasminogen activator receptor (uPAR) has been reported to predict SAP with a high diagnostic accuracy among patients in a tertiary center. The aim of the study was to compare the diagnostic utility of uPAR and other inflammatory markers as the predictors of the unfavorable course of AP in patients admitted to a secondary care hospital within the first 24 h of the onset of AP. The study included 95 patients, eight with a SAP diagnosis. Serum uPAR was measured on admission and in the two subsequent days. On admission, uPAR significantly predicted organ failure, acute cardiovascular failure, acute kidney injury, the need for intensive care, and death. The diagnostic accuracy of the admission uPAR for the prediction of SAP, organ failure, and ICU transfer or death was low to moderate and did not differ significantly from the diagnostic accuracy of interleukin-6, C-reactive protein, procalcitonin, D-dimer and soluble fms-like tyrosine kinase-1. In the secondary care hospital, where most patients with AP are initially admitted, uPAR measurements did not prove better than the currently used markers.

## 1. Introduction

Acute pancreatitis (AP) is one of the most common acute digestive tract diseases and, despite significant medical advancement in the last decade, it still poses a risk of life-threatening complications. In case of severe acute pancreatitis (SAP), mortality can reach 20–30% [1,2,3]. Recent studies indicate that in consequence of the systemic inflammatory response syndrome (SIRS) and multiple organ dysfunction syndrome (MODS) nearly half of deaths occur within the first week of AP [1,4,5]. These findings have been reflected in the 2012 revised Atlanta classification [6], which defines the early and the late phases of the disease. The early phase covers the period of one week from the onset of symptoms and is followed by the late phase lasting weeks or even months. The first 48 h are important for the further course of AP as adequate clinical management (including intensive fluid resuscitation) during the so-called therapeutic window can reduce the risk of complications [1,3,4,7]. This is especially important for patients developing MODS who should be referred to an intensive care unit (ICU) [7]. The diagnostic process to assess AP severity should take place in the first 48 h from the onset of symptoms. At present, it involves clinical assessment, imaging tests, multi-variable predictive scores (Ranson, APACHE II, Glasgow, BISAP) and laboratory testing [1,3,7,8]. Unfortunately, none of the above diagnostic tools appears flawless.

The laboratory tests that have been proposed for the assessment of AP severity include proinflammatory [tumor necrosis factor-α (TNF-α), interleukins (IL): IL-6, IL-1β, IL-18, IL-8] and anti-inflammatory (IL-4, IL-10, IL-1 receptor antagonist) mediators, soluble receptors (soluble TNF-α receptor II), microRNAs, adhesive molecules (soluble intercellular adhesion molecule 1), growth factors (hepatocyte growth factor, tumor growth factor-β1), procalcitonin, and acute phase proteins [C-reactive protein (CRP), serum amyloid A] [5,9,10,11]. However, many of the above markers can only be measured with enzyme-linked immunoassays rarely conducted in real time because of long procedures and high costs when not applied in series. At present, no single laboratory marker can be recommended for AP severity stratification, although in the clinical practice CRP measurements are often used [4]. Moreover, IL-6 and procalcitonin can be useful, as patients with SAP present with clearly higher concentrations in the first 48 h from the onset of symptoms: ≥300 pg/mL [12] and ≥3.3 ng/mL, respectively [9,10,13].

Patients with SIRS of either infectious or non-infectious origin present a severe endothelial dysfunction and hypercoagulability [14,15,16]. Endothelial dysfunction, together with tissue hypoperfusion, lead to gradual progression of organ failure [5,14,17]. The process is accompanied by the activation of coagulation and fibrinolysis, reflected in altered results of laboratory coagulation tests, e.g., high D-dimer concentrations [16,18].

One of the earliest fibrinolysis mediators is the urokinase-type plasminogen activator (uPA) [17]. Its ability to convert plasminogen into plasmin is potentiated by binding to the uPA receptor (uPAR, CD87) expressed on the endothelial cells, monocytes, macrophages, T-cells and granulocytes [17,19,20,21]. Besides being involved in fibrinolysis, uPAR assists in cell adhesion, migration, chemotaxis, immune activation, and tissue reconstruction [22,23]. The protein is bound to cell membranes by glycosylphosphatidylinositol anchor and may be shed or released into body fluids [23]. Soluble uPAR is present in plasma or serum, urine and cerebrospinal fluid [20,21,22,23]. Increased serum uPAR has been associated with various inflammatory states, particularly with sepsis and non-septic SIRS [17,20,21,22]. The correlation with severity of organ failure and mortality, together with the relative stability of plasma or serum concentrations, make uPAR a promising clinical biomarker in patients with SIRS [20,22,24,25], but so far has not been extensively studied in AP. Nonetheless, in 2017, Lipinski et al. [26] showed a very high diagnostic accuracy of plasma uPAR for the prediction of SAP and fatal AP among patients of a tertiary referral hospital.

The aim of our study was to assess the diagnostic usefulness of uPAR in the prediction of severe course of AP in a secondary care hospital setting, where most patients with AP are initially admitted. We were interested to know whether uPAR testing may help in the early (preferably within the first 48 h from the onset of AP symptoms) prediction of organ failure and, thus, the need for ICU transfer, and whether in this regard uPAR outperforms other single laboratory markers associated with AP severity.

## 2. Materials and Methods

The prospective observational study was designed to test whether the concentrations of uPAR measured in serum on the first days of AP allow the prediction of disease severity assessed during the hospital stay, and to compare uPAR with the other single laboratory tests used to predict AP severity. The study included adult patients admitted with AP, treated at the Surgery Ward, Complex of Health Care Centers in Wadowice, Poland (a secondary care hospital). Consecutive patients with a diagnosis of AP who were admitted to the Ward between March 2014 and December 2015 were assessed regarding eligibility for the study. Only patients with symptoms of AP lasting up to 24 h before hospital admission were included in the study. The exclusion criteria were chronic pancreatitis, known malignancy, and chronic liver disease. The patients provided written informed consent for the study. The study included patients who were able to provide an informed consent within a day of admission. The patients included in the study were asked to give three blood samples for laboratory tests, one sample on each of the first three days of the hospital stay. The demographic and clinical data were collected, including the etiology of AP, data on complications, severity and treatment of AP during the hospital stay. The main outcome variables were the organ failure during the first week of the hospital stay, the severity of AP defined according to the 2012 Atlanta classification (based on the entire course of the hospital stay) [6], and the ICU transfer or death during the hospital stay. The study protocol was approved by the Bioethics Committee of the Beskidy Medical Chamber (approval number 2014/02/06/1 issued on 6 February 2014).

The diagnosis of AP was based on the 2012 revised Atlanta classification [6], when at least two out of three of the following criteria were positive, i.e., typical clinical presentation of AP, serum amylase or lipase activity three times the upper reference value, and typical findings in dynamic computed tomography or magnetic resonance imaging or ultrasonography. The severity of AP was classified according to the 2012 Atlanta classification [6] as mild acute pancreatitis (MAP), moderately severe acute pancreatitis (MSAP) or severe acute pancreatitis (SAP). Organ failure was identified using the Modified Marshall Scoring System (MMSS) [6]. The diagnosis of acute kidney injury (AKI) was based on the Kidney Disease Improving Global Outcomes (KDIGO) criteria [27]. The diagnosis of acute respiratory distress syndrome (ARDS) was based on the Berlin criteria [28].

On admission (day 1), and on the two following days (day 2 and 3), blood samples were obtained from patients for routine blood tests and for measurements of uPAR, IL-6 and soluble fms-like tyrosine kinase-1 (sFlt-1). The routine blood tests included complete blood cell count, serum concentrations of urea, creatinine, bilirubin, CRP and procalcitonin, serum activities of amylase, lactate dehydrogenase (LDH), aspartate and alanine aminotransferases (AST, ALT), and plasma concentrations of D-dimer. The routine tests were conducted on the day of blood collection in the Central Laboratory, Complex of Health Care Centers in Wadowice, Poland using automatic analyzers: Sysmex XN (Sysmex Corporation, Cobe, Japan) for the blood counts, Cobas E411 (Roche Diagnostics, Mannheim, Germany) and Vitros 5600 (Ortho Clinical Diagnostics, Raritan, NJ, USA) for biochemistry and immunochemistry, and Coag XL (Diagon, Budapest, Hungary) for the coagulation testing. The performance of routine laboratory tests was assessed with daily internal quality control and regular external quality control (including Centre for Quality Assessment in Laboratory Medicine in Poland and Randox International Quality Assessment Scheme, RIQAS), in line with good laboratory practice. Additional serum samples for IL-6, uPAR and sFlt-1 assessment were aliquoted and frozen in −80°C. IL-6 and sFlt-1 were measured by electrochemiluminescence immunoassay (ECLIA) on Cobas 8000 analyzer (Roche Diagnostics, Mannheim, Germany) in the Diagnostic Department of University Hospital in Krakow, Poland. The minimum detectable doses were 1.5 pg/mL for IL-6 and 10 pg/mL for sFlt-1. The tests were calibrated according to the manufacturer’s instruction, the measurements in patients’ samples were run in series and were preceded by controls (PreciControl Multimaker, Roche, Mannheim, Germany) on two levels. For the IL-6 test, the intraassay precision was ≤6.0% and the interassay precision was ≤8.5%, and for the sFlt-1 test, the intraassay precision was ≤3.9 and the interassay precision was ≤5.6%, as reported by the manufacturer of the tests. Serum uPAR was measured with enzyme-linked immunoassay, using Quantikine Human uPAR Immunoassay reagent kit (R&D Systems, Minneapolis, MN, USA) in the Department of Diagnostics, Chair of Clinical Biochemistry at Jagiellonian University, Krakow, Poland. The minimum detectable dose of uPAR was 0.033 ng/mL, the reference range provided by the manufacturer was 1.195–4.415 ng/mL. Patients’ samples were tested for uPAR in duplicates, and in series; each run was calibrated according to the manufacturer’s instructions. The intraassay precision for the test was ≤7.5% and the interassay precision ≤5.6%.

Nominal data were reported as number (percentage of the group). Quantitative data were reported as mean and standard deviation (SD) or median, lower and upper quartiles (Q1; Q3), depending on normality of each variable’s distribution (as assessed with Shapiro-Wilk’s test). The contingency tables were analyzed with Pearson’s chi-squared test. In case of three groups, the whole contingency table was analyzed first, and then the pairwise comparisons were done using Pearson chi-squared test with Bonferroni correction. Due to the non-normal distribution of most quantitative variables, Kruskal-Wallis’s analysis of variance (with post-hoc comparisons using Siegel and Castellan method) was applied when three groups were compared and Mann-Whitney’s test when two groups were compared. Spearman’s rank order coefficient was used to assess correlations. Simple and multiple logistic regression was used to assess uPAR as a predictor of unfavorable course of AP. Receiver operating characteristic (ROC) curves were used to evaluate the diagnostic accuracy of the studied laboratory tests; the cut-off values were selected by maximizing the Youden index. The statistical tests were two-tailed, and the results were considered significant at *p* < 0.05. Statistica 12 software (Statsoft, Tulsa, OK, USA) was used for computations.

## 3. Results

The study included 95 patients with AP, 30 women, 65 men, with a mean (SD) age of 48 (16) years. According to the 2012 Atlanta criteria, 29 patients (31%) were diagnosed with MAP, 58 (61%) with MSAP and 8 (8%) with SAP. MAP, MSAP and SAP patients did not differ significantly in terms of age, sex, percentage affected with comorbidities, and AP etiology (Table 1). Average Ranson’s score was higher among patients with MSAP and SAP, while SIRS was common among both MSAP and SAP patients. The patients’ treatment (the need for surgery, nutritional support and ICU transfer) reflected the severity of AP. Four patients (4%) died—one in the early and three in the late phase of AP (Table 1).

On admission, the patients with SAP were characterized with significantly higher concentrations of urea, creatinine, glucose and IL-6 (Table 2). Other studied laboratory markers did not differ significantly between the MAP, MSAP and SAP patients on admission, although the median concentrations of inflammatory markers: Serum uPAR, CRP, procalcitonin and plasma D-dimer, as well as the median numbers of leukocytes were relatively high among patients with SAP. This was also observed in the case of endothelial dysfunction marker, i.e., sFlt-1 (Table 2).

**Figure 1:**
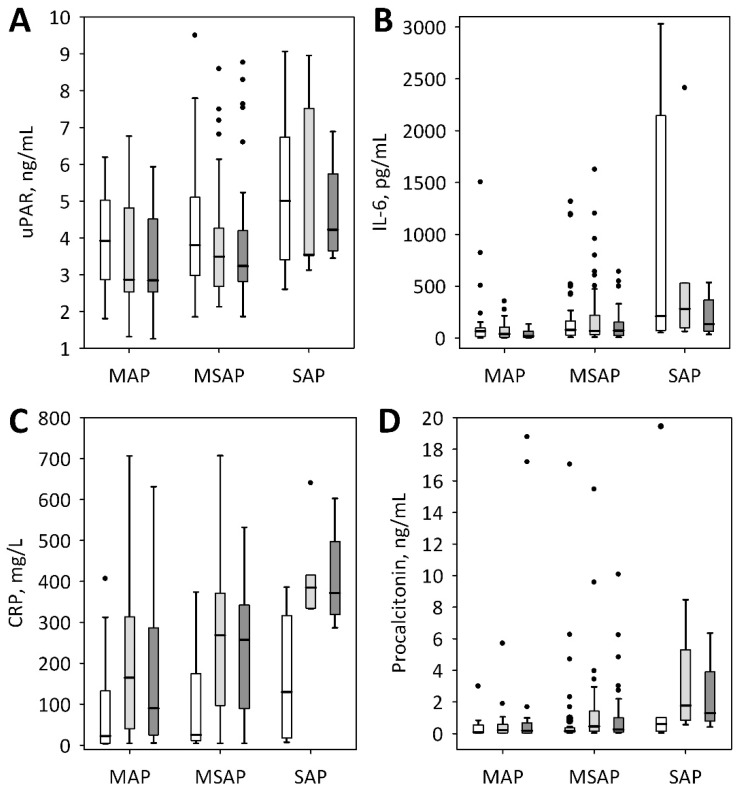
Serum concentrations of urokinase-type plasminogen activator receptor (uPAR) (**A**) among patients with mild (MAP), moderately-severe (MSAP) and severe acute pancreatitis (SAP) on admission (i.e., day 1 of the study, white bars), on day 2 (light grey bars) and day 3 (dark grey bars) of the hospital stay. Serum concentrations of interleukin-6 (IL-6) (**B**), C-reactive protein (CRP) (**C**), and procalcitonin (**D**) are shown for comparison. Data are presented as median, interquartile range (bars), non-outlier range (whiskers), and outliers (points).

Serum uPAR concentrations were significantly higher in women as compared to men, starting from the day of admission (Figure 1). No sex-related differences were observed for any other inflammatory markers (IL-6: *p* > 0.5; procalcitonin: *p* > 0.2; D-dimer: *p* > 0.1; white blood cells: *p* > 0.1; admission and day 3 CRP: *p* > 0.1), except for the day 2, when CRP was higher in men (median 315 in men versus 185 mg/L in women; *p* = 0.011). Also, no significant differences were observed between men and women regarding the severity of AP as defined according to the Atlanta classification (Table 1). Serum uPAR concentrations did not differ between patients with AP of various etiology (*p* = 0.7 on admission and day 2; *p* = 0.2 on day 3). No correlations were observed between uPAR and age. Patients with comorbid conditions (mainly ischemic heart disease) tended to present higher concentrations of uPAR, however, the difference was only significant on day 2 of the study: Median (Q1; Q3) was 3.77 (2.97; 5.03) ng/mL in those affected by comorbidities versus 2.93 (2.54; 3.88) ng/mL in the patients without comorbidities (*p* = 0.023).

**Figure 2:**
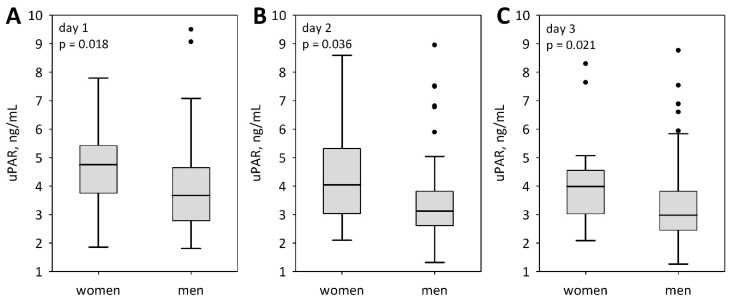
Sex-related differences in serum concentrations of uPAR among patients with AP during the first three days of hospital stay: On admission (day 1) (**A**), on day 2 (**B**), and on day 3 (**C**). Data are presented as median, interquartile range (bars), non-outlier range (whiskers), and outliers (points).

On days 2 and 3, serum uPAR concentrations were lower as compared to the admission levels, irrespective of AP severity (Figure 2A; *p* < 0.001 in MAP and MSAP groups; *p* = 0.039 in SAP). This contrasted with the other inflammatory markers, i.e., IL-6 (no significant changes), CRP (significant increase on day 2 and 3 comparing to admission concentrations), and procalcitonin (increase in MSAP and SAP patients) (Figure 1B–D). Although the patients with SAP tended to present higher serum concentrations of uPAR (Figure 1A), the differences between SAP and MAP or MSAP patients were non-significant throughout the study period. Also, no significant differences were observed between patients with edematous and necrotizing pancreatitis (*p* = 0.8 on day 1, *p* = 0.6 on day 2 and *p* = 0.8 on day 3 of the study). However, serum uPAR concentrations measured on admission significantly predicted organ failure (defined as two or more points in the modified Marshall scoring system, i.e., in line with the 2012 Atlanta classification), acute cardiovascular failure, acute kidney injury, the need for intensive care, and death (Table 3). Moreover, the maximum of the three measurements of uPAR (from admission until day 3 of the hospital stay) significantly predicted the same complications (Table 3). In the multiple logistic regression, the associations between both, the admission and the maximum uPAR and organ failure, acute cardiovascular failure, acute kidney injury, ICU transfer and death were all significant after adjustment for sex and the presence of comorbidities. Admission uPAR was a weak predictor of Ranson’s score of ≥3 points (Table 3) and it positively correlated with the Ranson’s score (R = 0.27; *p* = 0.012).

**Figure 3:**
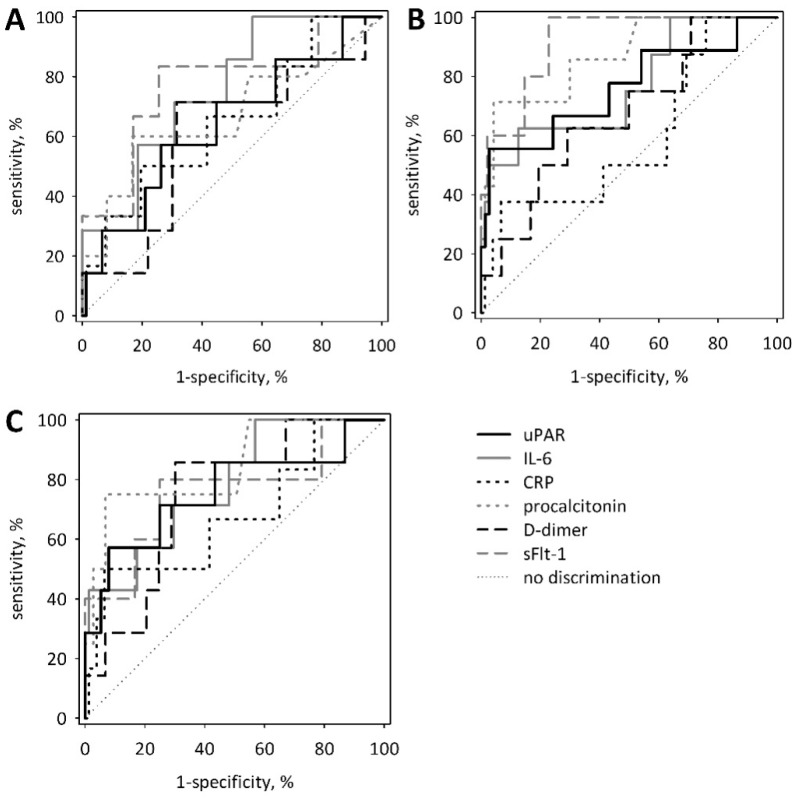
Receiver operating characteristic (ROC) curves for serum concentrations of uPAR on admission in prediction of SAP (**A**), vital organ failure (MMSS ≥2 points) (**B**), and ICU transfer or death (**C**). For comparison, ROC curves are shown for other proposed single biomarkers of AP severity measured on admission: Interleukin-6 (IL-6), C-reactive protein (CRP), procalcitonin, D-dimer, and soluble fms-like tyrosine kinase-1 (sFlt-1).

We compared the diagnostic accuracy of serum uPAR for the prediction of the unfavorable course of AP with the accuracy of other proposed single markers. On admission, no significant differences were observed in the areas under the ROC curves between uPAR and IL-6, CRP, procalcitonin, D-dimer and sFlt-1 in prediction of SAP, vital organ failure and ICU transfer or death (Figure 3). Again, in this analysis, uPAR was not a significant predictor of SAP (Table 4). However, uPAR significantly predicted organ failure and ICU transfer or death. The selected cut-off values (chosen by maximizing Youden index) allowed for high specificity at relatively low sensitivity (Table 4). For comparison, the areas under the ROC curves for IL-6, CRP, procalcitonin, D-dimer and sFlt-1 are shown in Appendix A (Table A1). The diagnostic accuracy of uPAR measured on day 2 and 3, or the maximum uPAR, were not significantly better as compared to the measurements on admission, although the area under the ROC curve for uPAR measured on day 3 of the study (72 h from the onset of symptoms) was significantly different from 0.5: 0.754 (0.595–0.913) (*p* = 0.002).

During the studied period, uPAR concentrations correlated with the other inflammatory markers, i.e., CRP (starting from day 2 of the hospital stay), procalcitonin, and IL-6 (Table 4). Positive correlation was observed between uPAR and sFlt-1 on admission and between uPAR and D-dimer on days 1 and 3. Serum uPAR negatively correlated with albumin (throughout the study) and hematocrit (starting from day 2). Moreover, uPAR correlated positively with bilirubin, aminotransferases and lactate dehydrogenase. No significant correlations were observed between uPAR and serum urea or creatinine concentrations (Table 5).

## 4. Discussion

The early identification of patients who are at risk of AP complicated by organ failure enables the provision of better care and allows for the proper allocation of intensive care resources. Despite intensive studies, we still lack laboratory markers which would allow for the early and accurate prediction of organ failure in AP. The widely adopted guidelines of the International Association of Pancreatology and the American Pancreatic Association recommend SIRS and the persistent (lasting ≥48 h) SIRS as early markers of severe AP, however, they also acknowledge other multiparameter scores and single laboratory markers (including CRP and procalcitonin). The guidelines emphasize the need for a repeated clinical assessment which takes into account risk factors (advanced age, comorbidities, obesity), clinical signs, and response to treatment [7]. At present, no single laboratory marker can be recommended for the early prediction of AP severity.

In the current study, we evaluated the soluble uPAR, a promising biomarker previously associated with mortality and severity of acute inflammatory conditions. Although serum uPAR measured within the first 24 h from the onset of AP symptoms significantly predicted the complicated course of AP, including the need for ICU transfer and death, its diagnostic accuracy did not appear better than that of other inflammatory markers (IL-6, procalcitonin, CRP) nor the markers associated with endothelial dysfunction and hypercoagulability (sFlt-1, D-dimer).

The soluble uPAR is considered to be a non-specific marker of inflammation, including SIRS, both the one that is related and unrelated to infection. Yu et al. [24] and Koch et al. [20] reported positive associations between circulating uPAR and proinflammatory cytokines (IL-6, TNF-α), procalcitonin and CRP. Such correlations were also observed in our study. Among patients treated in an ICU, high uPAR concentrations in serum or plasma have been associated with the severity of organ failure evaluated using the Sequential Organ Failure Assessment (SOFA), the Simplified Acute Physiology Score (SAPS) or the Acute Physiology and Chronic Health Evaluation (APACHE II) scores [17,20,21]. Circulating uPAR has been shown to predict mortality in patients with SIRS [17,29,30], cardiovascular diseases [31,32,33], ARDS [34] and sepsis [22,35]. Our results also show significant associations between the serum uPAR and the subsequent organ failure, the need for ICU treatment and death. In our patients, uPAR significantly predicted AKI and cardiovascular failure, but not ARDS.

We were able to identify only two previous reports regarding circulating uPAR concentrations in AP. Nikkola et al. [36] evaluated soluble uPAR as a predictor of alcoholic AP severity. Excessive alcohol consumption is the cause of nearly 40% of AP cases [2], and uPAR has been previously shown to be elevated in alcoholic liver disease [37]. Nikkola et al. [36] reported significantly higher plasma uPAR among patients classified as non-mild AP (MSAP and SAP) versus those with MAP, however, they used plasma samples collected from day 1 until day 4 from admission. They estimated that uPAR at a cut-off value of 5.0 ng/mL can predict non-mild AP with the sensitivity of 79% and the specificity of 78%; the area under the ROC curve was 0.81 (0.70–0.92) [36]. This diagnostic accuracy is higher than the observed in our study, however, our data are based on results obtained on admission, within the first 24 h from the onset of AP symptoms. Of note, in our study uPAR did not differ between patients with AP of various etiology, in particular, no differences were observed between those with alcoholic and biliary etiology. Lipinski et al. [26] studied plasma uPAR in patients with AP of various etiologies admitted to the tertiary referral hospital. They reported a very high diagnostic accuracy of plasma uPAR for the prediction of SAP: The area under the ROC curve was 0.993 (0.983–1.000), the diagnostic sensitivity 97% and the specificity 93% at the cut-off value of 4.75 ng/mL. In addition, they reported high diagnostic accuracy of uPAR for the prediction of multiple organ failure and death: Areas under the ROC curves of 0.951 (0.951–0.991) and 0.917 (0.882–1.000), respectively [26]. Moreover, uPAR >3.65 ng/mL allowed for the discrimination between MAP and MSAP with the 80% sensitivity and 92% specificity; the area under the ROC curve was 0.928 (0.883–0.972) [26]. The results are thus more optimistic than obtained by Nikkola et al. [36]. Obviously, our present study failed to replicate the results of Lipinski et al. [26]. There may be several reasons for this. Their study population differed from ours in the percentages of patients with SAP (26% versus 8%) and MSAP (29% versus 61%). The high percentage of patients with SAP in the group of Lipinski et al. [26] reflects the population of tertiary hospital patients. We can speculate that the severity of organ failure in that population may have also been higher than in our group, as the most severe cases are quickly transferred to tertiary centers. Consequently, the absolute number of patients with SAP reported by Lipinski et al. was substantially higher than in our group (33 versus 8 patients). The low number of patients with SAP in our group may in fact be responsible for the lack of a significant difference in uPAR concentrations between SAP and less severe AP, however, we also did not observe a significant difference between the MAP and MSAP patients, that was highly significant in the study of Lipinski et al. [26]. In our group, the percentage of patients with SAP is comparable to the recent reliable epidemiological data from Eastern Europe [2], but the high percentage of patients with MSAP in our group requires explanation. This is related to the arrangement of care in AP, often used in Polish secondary hospitals. We recruited patients of the surgical ward, while those with the predicted mild AP were admitted to the internal medicine/gastroenterology wards. In addition, the studies of Nikkola et al. [36], Lipinski et al. [26] and ours utilized different reagents and different samples for the measurements of uPAR: Nikkola et al. used plasma samples, but did not specify the anticoagulant. Lipinski et al. used EDTA-anticoagulated plasma, and we used serum samples. We measured uPAR with an enzyme-linked immunoassay, and although we followed good laboratory practice recommendations, the performance of the research assay could not be controlled as strictly as in the case of routine laboratory tests. Thus, the differences in laboratory methods may also be partially responsible for the differences in results of the three studies. This implicates the need for standardization of the laboratory methods before wider clinical use of uPAR.

In our study, uPAR correlated significantly with the laboratory markers of liver injury (bilirubin, transaminanases). Previous reports in AP did not explore such correlations, however, similar relationships were observed in the critically ill [20]. Koch et al. [20] observed significant correlations between uPAR and the markers of renal function, not present in our study. However, in our patients, high uPAR was a predictor of the subsequent AKI. Moreover, we studied the associations between the demographic variables and uPAR and observed significantly higher concentrations in women. This is in line with previous reports, including the study of Nikkola et al. [36] in alcoholic AP, but also the studies regarding other groups of patients [38,39].

The main and the most important limitation of our study is the low number of patients with SAP. Moreover, for the above-mentioned reasons, the percentage of patients with MAP and MSAP differ from the general epidemiological data. Therefore, we cannot draw definitive conclusions. Nonetheless, our study suggests that circulating uPAR measured on admission to a secondary care hospital allows for the prediction of the complicated course of AP with moderate diagnostic accuracy, comparable to other inflammatory, coagulation or endothelial markers, including the ones widely available in the clinical practice (CRP, procalcitonin, and D-dimer). Thus, it is too early to advocate for the use of uPAR in the early assessment of AP severity. More studies are needed that would allow the evaluation of the robustness of uPAR diagnostic performance in other health care settings. Also, the lack of laboratory standardization of uPAR measurements must be taken into account before wider clinical use of the marker.

## Figures and Tables

**Table 1:** Clinical characteristics of the study group according to the severity of acute pancreatitis (AP).

Characteristic	MAP (*n* = 29)	MSAP (*n* = 58)	SAP (*n* = 8)	*p*-Value
Male sex, *n* (%)	17 (59)	41 (71)	7 (88)	0.2
Mean age (SD), years	43 (16)	50 (16)	51 (20)	0.1
Pre-existing comorbidities, *n* (%)	10 (34)	27 (47)	5 (62)	0.3
Cardiac diseases, *n* (%)	5 (17)	20 (34)	5 (62)	
Diabetes, *n* (%)	0	6 (10)	2 (25)	
Dyslipidemia, *n* (%)	1 (3)	2 (3)	0	
Chronic kidney disease, n (%)	0	2 (3)	0	
Liver disease, *n* (%)	1 (3)	2 (3)	0	
Other comorbidities, *n* (%)	3 (10)	0	0	
Etiology				0.1
Biliary, *n* (%)	9 (31)	17 (29)	1 (12)	
Alcoholic, *n* (%)	12 (41)	11 (19)	6 (75)	
Hipertriglyceridemia, *n* (%)	1 (3)	4 (7)	0	
Other/idiopathic, *n* (%)	7 (24)	26 (45)	1 (12)	
Median Ranson score (Q1; Q3), points	2 (1; 3)	3 (3; 4)	6 (4; 7)	<0.001 ^a,b,c^
Median duration of hospital stay(Q1; Q3), days	10 (7; 12)	14 (10; 16)	26 (13; 41)	0.001 ^a,c^
SIRS in first 24 h, *n* (%)	18 (62)	49 (84)	7 (88)	0.047 ^c^
Early/late mortality, *n* (%)	0	0/2 (3)	1 (12)/1 (12)	0.006 ^a,b^
Therapeutic ERCP, *n* (%)	0	3 (5)	2 (25)	0.020 ^a,b^
Surgery, *n* (%)	0	3 (5)	4 (50)	<0.001 ^a,b^
Enteral feeding via nasojejunal tube, *n* (%)	0	4 (7)	6 (75)	<0.001 ^a,b^
Parenteral feeding, *n* (%)	0	1 (2)	2 (25)	0.001 ^a,b^
Transfer to ICU, *n* (%)	0	2 (3)	5 (62)	<0.001 ^a,b^

ERCP, endoscopic retrograde cholangiopancreatography; ICU, intensive care unit; MAP, mild acute pancreatitis; MSAP, moderately severe acute pancreatitis; n, number of patients; SAP, severe acute pancreatitis; SD, standard deviation; SIRS, systemic inflammatory response syndrome; Q1, lower quartile; Q3, upper quartile; *p*-value is reported for overall comparison between three groups (in Pearson chi-squared test or Kruskal-Wallis ANOVA), the letters in superscript indicate the results of post-hoc tests: ^a^ significant difference between the MAP and SAP groups in post-hoc comparison; ^b^ significant difference between the MSAP and SAP groups in post-hoc comparison; ^c^ significant difference between the MAP and MSAP groups in post-hoc comparison.

**Table 2:** The results of laboratory tests on admission according to the AP severity. Data are shown as mean (SD) or median (Q1; Q3).

Variable	MAP (*n* = 29)	MSAP (*n* = 58)	SAP (*n* = 8)	*p*-Value
Hematocrit, %	42 (5)	43 (6)	46 (7)	0.4
Albumin, g/L	38 (7)	35 (6)	36 (8)	0.6
Total calcium, mmol/L	2.13 (0.23)	2.15 (0.19)	1.92 (0.48)	0.5
Glucose, mmol/L	6.44 (5.61; 7.67)	8.17 (6.78; 9.33)	7.92 (7.22; 10.64)	0.002 ^a,c^
Urea, mmol/L	3.67 (2.83; 6.00)	4.67 (3.50; 6.00)	6.67 (5.00; 13.00)	0.015 ^a^
Creatinine, µmol/L	65.4 (59.2; 80.4)	69.8 (60.1; 87.5)	92.4 (75.6; 171.1)	0.033 ^a^
Bilirubin, µmol/L	23.4 (13.5; 38.5)	27.2 (13.8; 53.3)	29.1 (16.2; 36.9)	0.7
AST, U/L	59 (33; 209)	116 (55; 202)	122 (87; 166)	0.3
ALT, U/L	62 (43; 174)	133 (54; 299)	85 (49; 158)	0.3
LDH, U/L	553 (488; 810)	636 (507; 850)	1013 (737; 1294)	0.1
WBC, ×10^3^/µL	12.4 (9.5; 15.2)	13.1 (10.4; 16.2)	17.1 (10.3; 23.3)	0.4
Platelet count, ×10^3^/µL	199 (176; 231)	218 (165; 279)	227 (162; 292)	0.8
CRP, mg/L	22.7 (5.3; 132.4)	25.4 (11.9; 174.7)	129.6 (17.4; 316.7)	0.4
D-dimer, mg/L	1.49 (0.85; 2.19)	1.90 (1.00; 3.41)	2.76 (1.20; 3.39)	0.2
Procalcitonin, ng/mL	0.10 (0.05; 0.55)	0.17 (0.10; 0.36)	0.61 (0.14; 1.03)	0.1
uPAR, ng/mL	3.92 (2.86; 5.02)	3.81 (2.98; 5.10)	5.00 (3.41; 6.74)	0.4
Interleukin 6, pg/mL	64.7 (14.8; 95.7)	78.9 (27.8; 163.0)	210.7 (73.1; 21.5)	0.037 ^a^
sFlt-1, pg/mL	129 (119; 169)	140 (112; 154)	191 (155; 536)	0.1

ALT, alanine aminotransferase; AST, aspartate aminotransferase; CRP, C-reactive protein; LDH, lactate dehydrogenase; MAP, mild acute pancreatitis, MSAP, moderately severe acute pancreatitis; SAP, severe acute pancreatitis; sFlt-1, soluble fms-like tyrosine kinase-1; uPAR, urokinase-type plasminogen activator receptor; WBC, white blood cells; *p*-value is reported for overall comparison between three groups (in Kruskal-Wallis ANOVA), the letters in superscript indicate the results of post-hoc tests: ^a^ significant difference between the MAP and SAP groups in post-hoc comparison; ^b^ significant difference between the MSAP and SAP groups in post-hoc comparison; ^c^ significant difference between the MAP and MSAP groups in post-hoc comparison.

**Table 3:** Odds ratios (95% confidence intervals) for serum uPAR in prediction of unfavorable course of AP.

Dependent Variable	uPAR on Admission, per 1 ng/mL	Maximum uPAR, per 1 ng/mL
SAP (2012 Atlanta)	1.41 (0.92–2.17); *p* = 0.1	1.49 (0.94–2.37); *p* = 0.08
MSAP plus SAP (2012 Atlanta)	1.16 (0.84–1.60); *p* = 0.4	1.16 (0.86–1.58); *p* = 0.3
Persistent (≥48 h) SIRS	0.92 (0.69–1.22); *p* = 0.5	0.90 (0.69–1.18); *p* = 0.4
Ranson ≥ 3 points at 48 h	1.39 (1.01–1.89); *p* = 0.038	1.28 (0.96–1.71); *p* = 0.08
Organ failure (MMSS ≥ 2 points)	2.14 (1.33–3.46); *p* = 0.002	2.06 (1.30–3.26); *p* = 0.002
Cardiovascular failure	2.33 (1.34–4.08); *p* = 0.002	2.41 (1.29–4.50); *p* = 0.005
ARDS	1.01 (0.59–1.72); *p* = 0.9	1.19 (0.73–1.94); *p* = 0.5
AKI	1.78 (1.11–2.84); *p* = 0.015	1.77 (1.10–2.85); *p* = 0.017
ICU transfer	2.06 (1.24–3.43); *p* = 0.005	2.35 (1.27–4.35); *p* = 0.005
Death	1.82 (1.06–3.12); *p* = 0.027	2.25 (1.15–4.37); *p* = 0.015

AKI, acute kidney injury; ARDS, acute respiratory distress syndrome; ICU, intensive care unit; MMSS, modified Marshall scoring system; MSAP, moderately severe acute pancreatitis; SAP, severe acute pancreatitis; SIRS, systemic inflammatory response syndrome; uPAR, urokinase-type plasminogen activator receptor.

**Table 4:** Diagnostic accuracy of serum uPAR concentrations measured on admission for prediction of unfavorable course of AP.

Dependent Variable	AUC (95% CI)	*p* *	Cut-Off Value, ng/mL	Sensitivity, %	Specificity, %
SAP	0.641 (0.417–0.864)	0.2	5.004	57	75
Organ failure (MMSS ≥2 points)	0.761 (0.565–0.958)	0.009	6.736	56	97
ICU transfer or death	0.759 (0.536–0.983)	0.023	6.021	57	92

AUC, area under the receiver operating characteristic curve; CI, confidence interval; ICU, intensive care unit; MMSS, modified Marshall scoring system. * *p*-value in comparison with AUC = 0.5.

**Table 5:** Correlations of uPAR concentrations with selected laboratory markers during early stage of acute pancreatitis.

Variable	Day 1		Day 2		Day 3	
	R	*p*	R	*p*	R	*p*
Hematocrit	−0.11	0.3	−0.31	0.006	−0.27	0.022
Albumin	−0.32	0.014	−0.54	<0.001	−0.44	<0.001
Urea	0.16	0.2	0.10	0.4	−0.04	0.8
Creatinine	0.18	0.1	0.05	0.7	−0.05	0.7
Bilirubin	0.34	0.003	0.39	<0.001	0.41	<0.001
AST	0.49	<0.001	0.53	<0.001	0.39	0.001
ALT	0.37	0.001	0.37	0.001	0.30	0.009
LDH	0.46	<0.001	0.28	0.026	0.52	<0.001
WBC	−0.03	0.8	−0.03	0.8	0.19	0.09
CRP	0.19	0.1	0.29	0.012	0.42	<0.001
D-dimer	0.26	0.030	0.19	0.09	0.30	0.010
Procalcitonin	0.57	<0.001	0.54	<0.001	0.61	<0.001
Interleukin 6	0.25	0.027	0.35	0.002	0.54	<0.001
sFlt-1	0.48	<0.001	0.02	0.9	-	-

ALT, alanine aminotransferase; AST, aspartate aminotransferase; CRP, C-reactive protein; LDH, lactate dehydrogenase; sFlt-1, soluble fms-like tyrosine kinase-1; uPAR, urokinase-type plasminogen activator receptor; WBC, white blood cells.

## Appendix A

**Table A1:** The values of area under the receiver operating characteristic curve (AUC) with 95% confidence intervals (95% CI) for single laboratory markers proposed for the early assessment of severity of acute pancreatitis that were measured in the study and compared with uPAR. We provide AUC values estimated based on results obtained on the day of admission.

Laboratory Marker	AUC (95% CI); *p*-Value * for Prediction of		
	SAP	Organ Failure (MMSS ≥2 Points)	ICU Transfer or Death
IL-6	0.753 (0.590–0.917); *p* = 0.002	0.767 (0.578–0.956); *p* = 0.006	0.781 (0.610–0.953); *p* = 0.001
CRP	0.647 (0.412–0.882); *p* = 0.2	0.592 (0.373–0.810); *p* = 0.4	0.675 (0.425–0.925); *p* = 0.2
Procalcitonin	0.669 (0.378–0.961); *p* = 0.3	0.870 (0.729–1.000); *p* < 0.001	0.844 (0.627–1.000); *p* = 0.002
D-dimer	0.605 (0.377–0.901); *p* = 0.4	0.674 (0.485–0.863); *p* = 0.07	0.746 (0.582–0.909); *p* = 0.003
sFlt-1	0.770 (0.546–0.993); *p* = 0.018	0.921 (0.825–1.000); *p* < 0.001	0.758 (0.495–1.000); *p* = 0.054

* *p*-value in comparison with AUC = 0.5.
